# Retraction Note to: Treatment comparison of femoral shaft with femoral neck fracture: a meta-analysis

**DOI:** 10.1186/s13018-021-02748-0

**Published:** 2021-10-18

**Authors:** Yao Lu, Yakang Wang, Zhe Song, Qian Wang, Liang Sun, Cheng Ren, Hanzhong Xue, Zhong Li, Kun Zhang, Dingjun Hao, Yang Zhao, Teng Ma

**Affiliations:** Department of Orthopaedic Surgery, HongHui Hospital, Xi’an Jiaotong University, Xi’an, 710054 Shaan’xi Province China

## Retraction Note to: Journal of Orthopaedic Surgery and Research (2020) 15:19 https://doi.org/10.1186/s13018-019-1496-z

The Editor-in-Chief has retracted this article at the request of corresponding author Teng Ma. Following publication, it was found that five studies included in this systematic did not meet the inclusion criteria. The results of this review article are therefore unreliable. The authors have not responded to correspondence regarding this retraction notice.

